# MS Ana: Improving Sensitivity in Peptide Identification with Spectral Library Search

**DOI:** 10.1021/acs.jproteome.2c00658

**Published:** 2023-01-23

**Authors:** Sebastian Dorl, Stephan Winkler, Karl Mechtler, Viktoria Dorfer

**Affiliations:** † University of Applied Sciences Upper Austria, Bioinformatics Research Group, Softwarepark 11, 4232Hagenberg, Austria; ‡ Department of Computer Science, Johannes Kepler University Linz, Altenbergerstraße 69, 4040Linz, Austria; ¶ Research Institute of Molecular Pathology (IMP), Protein Chemistry, Campus-Vienna-Biocenter 1, 1030Vienna, Austria; § Institute of Molecular Biotechnology (IMBA), Protein Chemistry, Vienna Biocenter (VBC), Dr. Bohr-Gasse 3, 1030Vienna, Austria; ∥ Gregor Mendel Institute of Molecular Plant Biology of the Austrian Academy of Sciences (GMI), Dr. Bohr Gasse 3, 1030Vienna, Austria

**Keywords:** proteomics, bioinformatics, tandem mass spectrometry, spectral library search, peptide identification

## Abstract

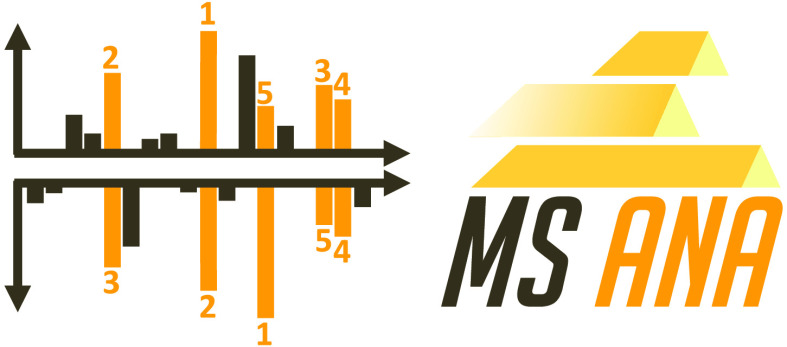

Spectral library search can enable more sensitive peptide identification in tandem mass spectrometry experiments. However, its drawbacks are the limited availability of high-quality libraries and the added difficulty of creating decoy spectra for result validation. We describe MS Ana, a new spectral library search engine that enables high sensitivity peptide identification using either curated or predicted spectral libraries as well as robust false discovery control through its own decoy library generation algorithm. MS Ana identifies on average 36% more spectrum matches and 4% more proteins than database search in a benchmark test on single-shot human cell-line data. Further, we demonstrate the quality of the result validation with tests on synthetic peptide pools and show the importance of library selection through a comparison of library search performance with different configurations of publicly available human spectral libraries.

## Introduction

Proteomics methods seek to measure the entirety of proteins in biological samples. Tandem mass spectrometry has become a premier technology to identify proteins in such complex mixtures.^1,2^ In a typical experiment proteins are measured indirectly from their peptides (bottom-up proteomics).^3^ Peptides are identified from tandem mass spectra by matching each spectrum to a peptide sequence based on its characteristic peptide fragment ion patterns.^4^

Algorithms for the identification of peptides from tandem mass spectrometry can be broadly categorized by how they construct their search space, that is, the total set of possible sequences that is considered when matching spectra to peptides. For instance, *de novo* search uses the search space of all theoretically possible amino acid sequences.^5^ The most common method is database search which limits the search space to only sequences found in a specific protein sequence database.^6−8^ Alternatively, spectral library search limits the search space to only peptides that have previously been measured with mass spectrometry.^9^

Spectral library search has theoretical advantages over database search. The database search space can grow exponentially when considering more and more sequence variations such as missed cleavage sites and post-translational modifications. A rapidly increasing search space leads to high processing times and higher probabilities for false positive identifications. Moreover, library search can use more of the information contained in a spectrum such as the intensity of fragment ion peaks and noncanonical fragment ions. Because of this, library search has been demonstrated to enable searching with higher sensitivity and higher specificity than database search.^10^

A number of software programs dedicated to spectral library search have been released such as SpectraST,^11^ Pepitome,^12^ MSPepSearch (derived from MSSearch^13^), ANN-SoLo,^14^ and COSS.^15^ However, library search has not become widespread for data-dependent acquisition experiments (spectral library methods are popular in data-independent acquisition^16^), as it is often held back by issues concerning the availability of high-quality libraries and result validation.

The creation of high-quality spectral libraries is challenging.^17,18^ On one hand, if the filter criteria are too strict then the coverage will be small and less identifications will be possible. On the other hand, if too many low-quality identifications are allowed in the library then these can propagate into library search results as false positives. Several sources have released large-scale spectral libraries such as the Global Proteome Machine Database (GPMDB^19^), the National Institute of Standards and Technology (NIST),^20^ MassIVE,^21^ PeptideAtlas,^22^ and PRIDE.^23^ However, few are able to regularly curate and maintain new libraries. MassIVE and NIST are the main providers for updated libraries using high-accuracy fragment ion HCD data. A solution for limited availability of libraries could be the application of predicted spectral libraries. Several approaches for the prediction of tandem mass spectra from sequences have been proposed.^24^ A variety of software tools are capable of predicting full spectral libraries from protein sequence databases such as INFERYS,^26^ MS2PIP,^27^ AlphaPeptDeep,^28^ and pDeep3.^29^

The accepted method for validation of results from database search is the target-decoy approach,^30^ but extending this method to spectral library search is not trivial.^31^ A major challenge is the generation of adequate decoy libraries. To fulfill the core assumptions of target-decoy the decoy spectra must be sufficiently realistic, that is, have similar properties to experimental spectra in terms of fragmentation patterns, number of explained peaks, signal-to-noise ratio, peak density, etc. A number of algorithms and advancements have been proposed to create decoy spectra.^31−34^ Some software tools have included the option to generate decoy libraries like SpectraST^11^ or the CompOmics Spectral Library Searching Tool (COSS).^15^ Still, the added complexity of decoy spectra generation means that few readily available solutions exist for decoy library generation.

We present MS Ana, a new spectral library search engine that enables high performance searches for various library configurations including fully predicted spectral libraries and enables target-decoy validation through its own decoy library generation algorithm. To demonstrate the performance of MS Ana, we first perform validation experiments using synthetic peptides. Then, we compare the performance on benchmark data sets using various public human libraries. Finally, we show the performance in a typical experimental setting.

## Methods

### Spectral Library Search Algorithm

The MS Ana Spectral Library Search Engine identifies peptides in tandem mass spectra by computing spectrum-to-spectrum matches between input spectra and a library of previously identified spectra.

Before searching, input spectra and library spectra are subjected to equivalent preprocessing steps. First, unspecific fragment ions are removed from the spectrum which includes all ions derived from the precursor as well as low mass ions.^35^ Then, peak picking is performed: For every spectrum, the lowest intensity peaks in each 100 *m*/*z* window are removed until each window has at most *m* peaks where *m* is the peak picking depth. Finally, the absolute peak intensities in the spectrum are scaled such that the sum of intensities is constant for all spectra.

The MS Ana score *S*(*s*_I_, *s*_*L*_) between an input spectrum *s*_*I*_ and a library spectrum *s*_*L*_ consists of a probabilistic score *P*(*s*_*I*_, *s*_*L*_) based on probabilities to match peak masses and intensity ranks as well as a quantitative score *Q*(*s*_*I*_, *s*_*L*_) based on the ratio of matched peak intensity from specific ion series. The MS Ana score is defined as
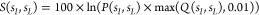
1where the probabilistic score *P*(*s*_*I*_, *s*_*L*_) is obtained from three different p-values combined by Fisher’s Method^36^ as
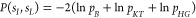
2where the first part of the probabilistic score is based on a cumulative binomial distribution function. The p-value *p*_*B*_ represents the probability of matching as many or more peaks by chance and is calculated as
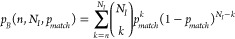
3where *n* is the number of matched peaks between *s*_*I*_ and *s*_*L*_. *N*_*I*_ is the total number of peaks in *s*_*I*_ after preprocessing. *p*_*match*_ is the probability to match one peak by chance and is given by the fraction of the *m*/*z* range that is covered by the peaks in *s*_*L*_ and the total mass window in *s*_*I*_ (from first peak to last peak). The calculation of *p*_*match*_(*s*_*I*_, *s*_*L*_) and its implementation are taken from the scoring function used in the MS Amanda Database Search Engine.^8^ As such, the *p*_*B*_ portion of the MS Ana score does not take peak intensities into account.

The second part of the probabilistic score is based on the Kendall-Tau rank correlation^37^ of intensity ranks between the matched peaks in *s*_*I*_ and *s*_*L*_. The p-value *p*_*KT*_ represents the probability of obtaining the same or a better correlation of peak ranks by chance and is calculated as
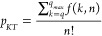
4where *q* is the number of neighbor interchanges required to transform the list of ranks for the matched peaks in *s*_*L*_ into the list of ranks for the matched peaks in *s*_*I*_ with *q*_*max*_ being the maximum possible *q* if the lists have minimal correlation. *n* is the number of matched peaks between *s*_*I*_ and *s*_*L*_. *f*(*q*, *n*) is Kendall’s rank correlation frequency distribution, that is, the frequency total for each unique *q* in case of *n* ranks. In MS Ana, *f*(*q*, *n*) is obtained using an exact calculation of the frequency distribution for permutations of up to *n* = 170 matched peaks.^38^ A similar use of Kendall-Tau rank correlation for spectrum-to-spectrum matching was previously described by Dasari et al.^12^

The third part of the probabilistic score is based on a cumulative hypergeometric distribution. The p-value *p*_*HG*_ represents the probability to match as many or more peak intensity ranks among the matched peaks in *s*_*I*_ and *s*_*L*_ and is calculated as
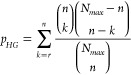
5where *n* is the number of matched peaks between *s*_*I*_ and *s*_*L*_ and *r* is the number of matched peak pairs where both peaks have precisely the same intensity rank in their respective spectra. *N*_*max*_ is the maximum number of total peaks given as max (*N*_*I*_, *N*_*L*_)

Finally, the quantitative score *Q*(*s*_*I*_, *s*_*L*_) is obtained from the fraction of relevant fragment ions that were matched in *s*_*L*_ and is calculated as
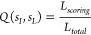
6where *L*_*scoring*_ is the sum of intensities for scoring peaks in *s*_*L*_. We define a scoring peak as a peak in *s*_*L*_ that was matched to a peak in *s*_*I*_ and was also annotated as part of a relevant type of fragment ion series. By default, the scoring ion series are y-ions, b-ions, as well as any internal fragments that derive from them. *L*_*total*_ is the total sum of peak intensities in *s*_*L*_.

MS Ana exposes all of the described subscores as well as the absolute and relative intensities for all matched ion series in its output to be accessible for postprocessing methods such as Percolator.

### Decoy Library Generation

MS Ana can generate new decoy spectral libraries from any target library by using an adapted version of the shuffle-and-reposition approach.^32^ In brief, a new decoy spectrum is generated from an existing library spectrum by moving fragment ion peaks such that the decoy is sufficiently different but still constitutes a realistic tandem mass spectrum.

MS Ana first generates a decoy sequence from the peptide sequence associated with the library spectrum by rearranging the amino acids in the sequence. The C-terminal and N-terminal amino acids are kept fixed, the remaining amino acids are randomly shuffled. Any modifications attached to specific amino acid positions are moved accordingly. To ensure that amino acid positions in the new sequence have been sufficiently shifted we calculate the Kendall-Tau rank correlation *tau*(37) on the positional vectors. If *tau* > 0.5 the sequence is shuffled anew until the threshold is met. If shuffling is unsuccessful after *len*(*sequence*)^2^ trials, no decoy is created which is a highly unlikely outcome (for instance, did not occur for the MassIVE Human HCD library).

Using the target sequence and decoy sequence, MS Ana calculates the mass shift δ for every annotated fragment ion in the spectrum as
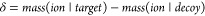
7where *mass*(*ion*|*target*) and *mass*(*ion*|*decoy*) are the theoretical masses of the ion given the target sequence or decoy sequence, respectively. Following that, all annotated peak masses are shifted by their δ to populate the decoy spectrum (the peaks retain their respective intensities). Peaks that would shift out of the detectable *m*/*z* range are discarded. Peaks without annotation are copied from target spectrum to decoy spectrum as is.

The generation of decoy spectra by shuffle-and-reposition requires a high ratio of annotated fragment ion peaks. For this purpose, MS Ana implements an adapted version of the expert system for annotation of tandem mass spectra described by Neuhauser et al.^39,40^ In brief, starting with the target sequence, a list of theoretical fragment ions of all common ion series is created. The fragment ions are matched to peaks in the library spectrum. Subsequently, the successfully matched annotations are used to create a derived list of theoretical fragment ions with additional neutral losses or internal fragmentation events. The new ions are matched and the process is repeated until no more peaks can be annotated. In case two annotations are assigned to one peak, a priority system is used to ensure that the most common fragment ion is matched. The created peak annotations are written into the target spectral library and are subsequently used during searching for the calculation of the quantitative part of the MS Ana score (see description of eq 6).

The creation of decoys is directly based on annotated peaks and the annotation of peaks in turn is done at a specific match tolerance. Because of this, the tolerance should be adjustable and match the fragment tolerance used for library searches. Starting an MS Ana library search with fragment mass tolerance or preprocessing parameters that do not match the existing decoy library will prompt the creation of new annotations and decoys.

### Synthetic Peptide Pools for Validation

Zolg et al.^41^ have assembled a large collection of synthetic peptide data measured by mass spectrometry, and results are available in full from the ProteomeXchange PRIDE archive^23^ via data set identifier PXD004732. Each individual run includes the combined synthesis products for a pool of 1,000 peptides and 88 quality control peptides. When reanalyzing these data sets, we can expect to predominantly identify sequences from this pool of peptides. We use this synthetic peptide pool data to prepare validation tests for the identification algorithms.

For the search results we first estimate the normal false discovery rate (FDR) from the target-decoy approach^42^ as *FDR* = *decoy hits*/*target hits* where target hits and decoy hits are the number of spectrum-level identifications above the score threshold in the target and decoy search space, respectively. Following that, we calculate an additional synthetic false discovery rate as *synthetic FDR* = *incorrect hits*/(*incorrect hits* + *correct hits*). We define a correct hit as a target hit that either matches a sequence in its synthetic peptide pool or matches a subsequence of a sequence in its synthetic peptide pool. The inclusion of subsequences might suggest that many more correct matches are possible. However, these matches are still restricted by the sequences available in the synthetic peptide pool which includes only tryptic peptides with 7 amino acids or more.

Because each peptide pool only includes sequences of very similar length using individual files is not representative of a typical mass spectrometry experiment. We combined 15 files from the 126 HCD runs given for the proteotypic set of pools. The resulting test file had 780,663 spectra and a total pool of 15,088 peptide sequences. This specific list of files was selected to match the overall distribution of peptide lengths (see Supplementary Figure S1). To investigate the robustness of the synthetic FDR approach we also selected an alternative set of 15 files and repeated the validation test. The alternative test file included 778,674 spectra (see Supplementary Figure S1).

### Benchmark Data and Search Parameters

All mass spectrometry input files for analysis were downloaded from the ProteomeXchange PRIDE archive^23^ (see Supplementary Table S1 for exact identifiers and file names). The .raw files were imported into analysis workflows within Proteome Discoverer (Version 3.0.0.757, Thermo Fischer Scientific, Bremen; see Supporting Information for workflow files). After searching, .msf result files were extracted and processed further. Percolator^43^ and INFERYS^26^ were used for rescoring of identifications after search via their respective nodes in Proteome Discoverer using standard parameters.

MassIVE^21^ human peptide spectral libraries were downloaded from their Web site (massive.ucsd.edu) in .splib format (accessed May 2022). We used the *in vivo* HCD library and synthetic HCD library from the full release of the MassIVE Knowledge Base (Version 2.0.15).

NIST libraries of peptide tandem mass spectra are available from the National Institute of Standards and Technology Web site (peptide.nist.gov). We used the consensus human HCD libraries (accessed May 2022) “Library 1 (Best)” and “Library 2 (Good)”. Library files were downloaded as .msp format.

The proteome-wide *Arabidopsis thaliana* HCD spectral library^44^ was downloaded from ProteomicsDB^45^ (proteomicsdb.org, accessed June 2022) in the .msp format.

A predicted spectral library was created from the canonical Uniprot Swissprot human FASTA database (accessed May 2022) using the INFERYS^26^ platform’s predict spectrum library dialogue in Proteome Discoverer. Tryptic spectra were predicted using HCD activation type and a collision energy of 28. The predicted spectra had a peptide length of 7 to 30, precursor charge state of +2 to +4, as well as maximum missed cleavage sites of 1. Static modification of carbamidomethyl at cystein was selected as well as oxidation of methionine as dynamic modification.

All spectral libraries were imported into Proteome Discoverer to be used in analysis workflows (see Supplementary Table S2 for an overview of all library files).

MS Ana spectral library searches used a precursor mass tolerance of 10 ppm and fragment mass tolerance of 20 ppm. Preprocessing parameters were set to peak picking depth of 15 peaks per 100 *m*/*z* and annotation of all ion series in HCD fragmentation mode. Also the standard mode was selected for ion scoring which scores y-ions and b-ions, as well as internal fragments. All MS Ana decoy libraries were generated using the random sequence algorithm.

MSPepSearch is the spectral library search program developed and maintained by NIST and originally based on MSSearch.^13^ MSPepSearch search runs were done using the MSPepSearch node available in Proteome Discoverer. Searches used a precursor mass tolerance of 10 ppm and a fragment mass tolerance of 20 ppm. MSPepSearch itself does not generate decoy spectra. Instead, decoy spectrum libraries were created on library import in Proteome Discoverer. Parameters for library generation were: HCD activation type and fragment mass tolerance of 20 ppm for NIST and ProteomicsDB libraries. MassIVE spectral libraries used a 100 ppm fragment mass tolerance because decoy library creation in Proteome Discoverer could not be completed at 20 or 50 ppm. Proteome Discoverer implements the reverse decoy library generation algorithm.^31^

Sequest^6^ was used as a database search engine using the multicore optimized version Sequest HT implemented in Proteome Discoverer. Canonical Uniprot Swissprot human FASTA database (accessed May 2022) was used for runs on human data together with the Universal Contaminants FASTA.^46^ Searches used a precursor mass tolerance of 10 ppm, fragment mass tolerance of 0.02 Da, and full tryptic digestion with a maximum of one missed cleavage. Modifications included carbamidomethyl at cystein (static) and oxidation of methionine (dynamic).

## Results and Discussion

### Validation of Search Engines with Synthetic Peptide Data

**Figure 1:**
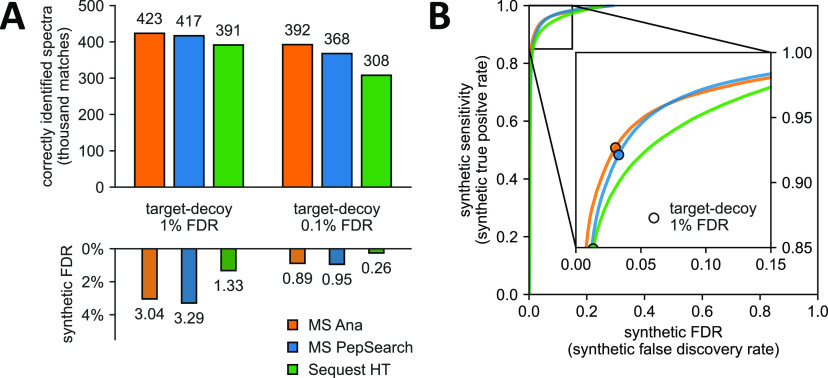
Spectral library search provides greater sensitivity during identification: Validation of identification algorithms on synthetic peptide data. Spectral library search and database search were tested on a combined HCD Orbitrap Fusion data set of 780,663 spectra for a pool of 15,000 synthetic peptides obtained by Zolg et al.^41^ (A) Comparison of correctly identified spectra. After validation by target-decoy search only matches that are in the list of synthetic peptides are retained as correct identifications and a new synthetic FDR is calculated accordingly. (B) Comparison of synthetic FDR and sensitivity for the search results. True positives are those spectra which were correctly identified from the list of synthetic peptide pool sequences. The specific threshold for target-decoy FDR 1% is highlighted.

To validate the performance of MS Ana spectral library search and its decoy generation algorithm, we devised a test setup on a data set of synthetic peptides originally obtained by Zolg et al.^41^ (Figure 1). Using the list of synthetic peptide sequences as ground truth, we can separate the final results into correct (true positive: (sub)sequence is in the synthetic peptide pool) and incorrect identifications (false positive: (sub)sequence is not in the synthetic peptide pool). At 1% target-decoy FDR the MS Ana results contained 423,423 spectra (54.24% of the data set) that were matched to a correct synthetic peptide sequence (Figure 1A) which was an increase of 6,591 and 32,065 true positive identifications compared to MSPepSearch and Sequest, respectively (see Supplementary Table S3 for more details).

Based on the number of 13,281 false positive identifications, we calculated a synthetic FDR of 3.04% for MS Ana which was better than that of MSPepSearch but worse than that of Sequest. Compared to Sequest, the target-decoy approach with MS Ana underestimated the FDR by 1.72%. We argue that this is acceptable when compared to the increase of true positive identifications of 8.2% for MS Ana. Furthermore, MS Ana performs better at the stricter target-decoy FDR threshold of 0.1% with an increase of correctly identified spectra of 24,568 (6.7%) and 84,427 (27.4%) compared to MSPepSearch and Sequest, respectively.

In all test runs the FDR values estimated by the target-decoy approach underestimated the FDR calculated from synthetic peptide pool sequences. We can attribute this to technical variations in the peptide synthesis. Zolg et al.^41^ reported an average recovery of 95% in the HCD synthetic peptide pools but also found many synthesis byproducts. For our test we only accounted for truncated synthesis products by allowing all subsequences of correct peptides as true positive identifications. Furthermore, results show that both spectral library search engines underestimated the FDR more than database search. We suggest that this is caused by the increased difficulty of generating good decoy spectral libraries compared to decoy database creation by sequence reversion.^31^ This highlights the need for strict validation of FDR control for newly proposed library search algorithms.

Figure 1B shows the sensitivity and FDR curves for the true positive and false positive identifications. This enables us to compare the performance at all possible validation thresholds. Both library search methods maintained higher sensitivity than database search at relevant validation thresholds. Furthermore, MS Ana consistently showed the highest sensitivity for a synthetic FDR below 5% which is the area that is most important for typical experiments because it includes the common target-decoy validation threshold of 1% FDR. These results demonstrate the greater identification performance of spectral library search.

To investigate the robustness of the test results, we repeated all searches on an alternative data set drawn from the same collection of synthetic peptide data (see Supplementary Figure S2). In this case, the calculated synthetic FDR (at 1% target-decoy FDR) was higher: 3.56% for MS Ana, 3.70% for MSPepSearch, and 1.43% for Sequest. At 1% target-decoy FDR MS Ana found 1.7% and 6.3% more true positive identifications than MSPepSearch and Sequest, respectively. The comparison of results for the different search engines was consistent between the two data sets.

This test setup was designed to match the search space of all engines as close as possible to each other and to the data. Both spectral library search engines used the MassIVE HCD synthetic target library which was partially constructed from the same synthetic peptide pool data we searched against. Sequest was using a custom FASTA file that was created by extracting all peptide sequences in the same MassIVE target library. Because of the matched libraries and database we can attribute the difference in identifications directly to search performance instead of any differences in search space.

One advantage of spectral library search is its ability to more reliably identify lower quality spectra compared to database search.^47^ This presents a challenge for designing validation experiments because test data sets tend to favor high-quality spectra. Tests of search algorithms often use ground truth data sets for which the correct identification is precisely known for each spectrum.^31,48^ When the data set is constructed from spectra that can be identified with high confidence the result necessarily includes more high-quality spectra, that is, spectra with higher signal-to-noise ratio and more explained peaks. We suggest that this issue can be addressed by using full measurement runs of synthetic peptide pools. This approach enables us to differentiate correct from incorrect identifications while retaining a distribution of low- and high-quality spectra more similar to that of standard mass spectrometry experiments.

Because the different search engines use different approaches for both search and decoy generation these aspects should be tested explicitly. Otherwise we might run into the risk of overstating the value of a search engine if the decoy library is not adequately constructed. In our tests MS Ana used its own decoys generated by an adapted random sequence decoy spectrum algorithm while MSPepSearch used a version of the reverse sequence decoy spectrum algorithm implemented in Proteome Discoverer. Our results show that both combinations are effective with MS Ana achieving more correct identifications and a synthetic FDR closer to the target-decoy FDR. These findings match well with the results obtained by Zhang et al.^31^ who compared reverse and random decoy spectral libraries on a different set of the same synthetic peptide data. They found a difference of less than 2% for the two different decoy generation approaches. This also suggests that the increased identifications for MS Ana result from our refined spectrum-to-spectrum matching score which can use more information from the spectra. We propose this setup using synthetic peptide data as a complementary approach to the tests described by Zhang et al.^31^

### Comparison of Spectral Library Performance

**Figure 2:**
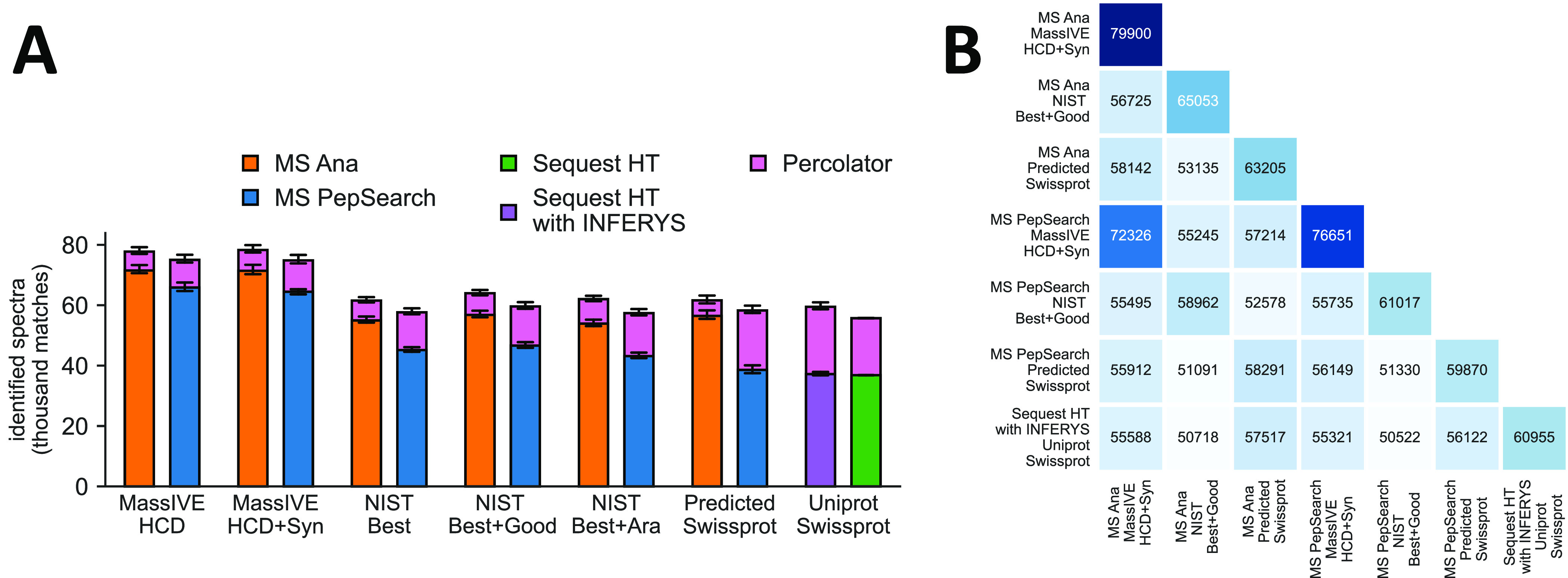
Current human libraries can outperform database search: Comparison of spectral library search results using publicly available libraries on a benchmark data set from a K562 cell-line digest measured on Orbitrap QE HF-X by Van Puyvelde et al.^49^ (A) Overview of identification results using different combinations of spectral libraries (orange and blue) compared to databse search (green and purple). Additional identifications from using Percolator^43^ on the results are added on top of the bars. All results filtered to 1% FDR. Error bars indicate standard deviation (*n* = 3 replicates). (B) Overlap of identified spectra on the first replicate using selected configurations for each algorithm. Entries indicate the number of peptide-to-spectrum identifications that match exactly between two results.

A major challenge for spectral library search is the need for high-quality libraries. In the case of human samples, some good candidates have been released in recent years. We compared the performance of MS Ana using several different combinations of publicly available human HCD spectral libraries. For this test we used a recently published benchmark data set obtained by Van Puyvelde et al.^49^Figure 2 shows the results comparing identified spectra at 1% target-decoy FDR for the different spectral library searches and database search (see Supplementary Table S5 for more details).

The best result was achieved by MS Ana using the current HCD human library from the MassIVE 2.0.15 Knowledge-Base^21^ (”MassIVE HCD”) combined with their HCD human synthetic spectral library (”MassIVE HCD+Syn”). In this configuration MS Ana identified on average 4.6% more spectra than MSPepSearch and 31.8% more spectra than database search with Sequest (using INFERYS). However, the addition of the synthetic spectra to the search only increased the number of identifications by 0.7% for MS Ana and even decreased it by 0.2% for MSPepSearch while at the same time widening the search space by 77% and increasing processing times accordingly.

NIST is another source for high-quality human HCD spectral libraries.^20^ We tested the NIST consensus human HCD library (updated May, 2020) designated as ”Best” which includes only high-quality spectra and no missed cleavages (”NIST Best”) as well as using it in combination with the NIST library with designation “Good” which includes medium-quality spectra and missed cleavages (“NIST Best+Good”). Searches with the NIST library produced fewer results than those with MassIVE HCD, reducing identifications by 20.8% and 23.1% for MS Ana and MSPepSearch, respectively. This gap narrowed to 17.7% for MS Ana and 20.6% for MSPepSearch when using the NIST Best+Good library at the cost of increasing total search space by 50%. This discrepancy is perhaps not surprising given the difference in proteome coverage. The initial MassIVE human spectral library reported a total proteome coverage of 54%,^21^ whereas the tested NIST libraries cover 27.9% (“NIST Best”) and 32.2% (“NIST Best+Good”) of the human proteome (as per the NIST Web site peptide.nist.gov).

Additionally, we directly tested the influence of spectral library size using an out-of-sample spectral library. The NIST Best library was combined with an *Arabidopsis* HCD spectral library^44^ (“NIST Best+Ara”). This increased the library size and total search space by 121% high-quality nonhuman spectra. Consequently, this had little impact on library search results. MS Ana identifications increased by 0.7% while MSPepSearch results decreased by 0.2%. These results demonstrate that the sensitivity of spectral library can make the search robust even when a large number of “wrong” spectra are included in the spectral library.

Moreover, we tested the library search performance using a fully predicted spectral library. The INFERYS^26^ platform in Proteome Discoverer was used to predict spectra for a tryptic digest of Uniprot Swissprot resulting in a library of 5,253,666 total entries (”Predicted Swissprot”). Performance of the predicted library was comparable to the NIST libraries (average difference of less than 1% identifications for MS Ana between NIST Best and Predicted Swissprot). Interestingly, the MS Ana search using the predicted spectral library only shows an average increase of 9.1% identifications from application of Percolator compared to the 50.7% for MSPepSearch. We speculate that the predicted spectra interact positively with the weighting of ion series in the MS Ana scoring function since only y-ion and b-ion series are predicted by INFERYS.

MS Ana identified more spectra than MSPepSearch in all configurations while consistently producing a higher overlap in identifications with the database search results from Sequest (compare results for the last row in Figure 2B). The greatest overlap of identifications between library search and database search was observed when using the predicted spectral library.

Notably, the increase of identifications from MS Ana to MSPepSearch is more pronounced before rescoring with Percolator. The effect is the largest for the predicted spectral library. This could make MS Ana more attractive in cases where Percolator cannot be used or for the purpose of using additional rescoring tools. INFERYS, for instance, which uses predicted spectra for rescoring of database search results, could theoretically also be applied to results from spectral library search. While gains from using INFERYS in this test case were only 5% the authors have reported that additional identifications of up to 50% are possible.^26^

Our tests demonstrate the high performance that spectral library search can achieve on human data using currently available libraries. While there are significant differences in the number of identified spectra depending on the choice of library (an increase of 26.2% identifications from the worst to best performing library with MS Ana), MS Ana was able to outperform database search in all tested configurations producing up to 31.8% more identifications than Sequest with INFERYS rescoring. As the quality of public libraries increases and proteome coverage rises we can expect the performance of library search to improve further. We also showed that predicted libraries can already perform similar to curated ones for human data. Consequently, spectral library search on data from nonmodel organisms will benefit even more from the use of predicted libraries which MS Ana can use to great effect.

### Single-Shot Data Dependent Acquisition Benchmark Results

**Figure 3:**
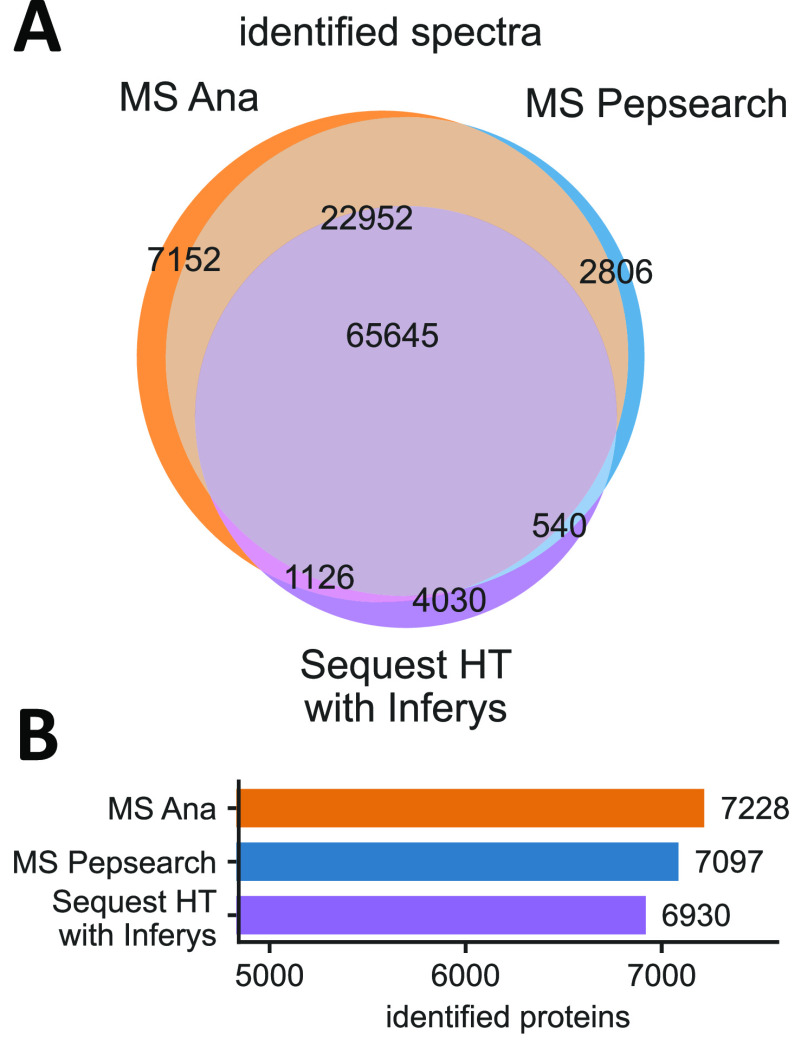
Spectral library search increases identifications in practice. Benchmark of identification algorithms on MCF-7 human cell-line data from a single shot DDA run using 2 h Microflow LC-MS/MS gradient and Q Exactive Orbitrap HF-X instrument measured by Bian et al.^50^ Search results of the first replicate comparing spectral library search using MS Ana (orange) or MSPepSearch (blue) on MassIVE Human HCD library with database search using Sequest (purple) on Uniprot Swissprot. (A) Venn diagram showing the overlap of peptide-to-spectrum matches between the different identification algorithms (1% FDR using Percolator^43^). (B) Number of identified proteins from result (1% Protein-level FDR applied after filtering by Percolator).

To demonstrate the performance of MS Ana for general use, we tested library search in a setting more closely resembling a routine mass spectrometry experiment. We chose to test on a data set of single-shot data dependent acquisition experiments originally measured by Bian et al.^50^Figure 3 shows the results for reanalyzing the first replicate, a 2 h gradient sample from the MCF-7 human cell-line experiments (see Supplementary Table S6 for more details). This search was using the MassIVE HCD human spectral library which was the best performing single-file spectral library from earlier tests.

MS Ana matched 96,875 spectra (59% of total) at 1% FDR which was an increase in identifications of 4,932 (5.4%) and 20,602 (35.8%) compared to MSPepSearch and Sequest, respectively. MS Ana also produced the most unique identifications (7,152) (Figure 3A). Both library search engines showed high agreement in identifications with 88,597 total matches being the same for MS Ana and MSPepSearch. Of these, 65,645 identifications overlapped between all three search engines. Furthermore, we obtained protein identifications from the results using the standard consensus workflow in Proteome Discoverer (Figure 3B). At 1% protein FDR MS Ana identified 7,228 proteins which was 131 (1.8%) and 298 (4.3%) more than MSPepSearch and Sequest, respectively.

The average number of identified proteins by database search with Sequest was 6,924 which matches closely to the 6,946 proteins originally reported by Bian et al.^50^ They were using database search followed by rescoring with Prosit,^25^ whereas we used rescoring with the INFERYS^26^ platform which is directly derived from Prosit.

Additionally, we performed an analogous benchmark test on data from a recent study using a timsTOF mass spectrometer. We reanalyzed triplicate data sets from human cardiac tissue originally measured on a Bruker timsTOF Pro by Aballo et al.^51^ Bruker files were converted to .mgf using the PASEF preset in MSConvertGUI^52^ (Version 3.0.22317, default parameters). Search and postprocessing workflows were equivalent to the benchmark on single-shot data dependent acquisition. On the timsTOF data MS Ana identified on average 4,345 proteins at 1% FDR which was 102 (2.4%) and 468 (9.4%) more than MSPepSearch and Sequest, respectively (see Supplementary Figure S3 and Supplementary Table S7 for more details).

Our tests demonstrate that spectral library search combined with publicly available libraries can improve identification results for a common mass spectrometry setup. Single-shot approaches in particular can suffer from limited proteome coverage^50^ enabling them to benefit greatly from the increased sensitivity provided by spectral library search.

## Conclusion

We presented MS Ana, a new spectral library search engine for use with high-accuracy tandem mass spectrometry data from data dependent acquisition proteomics experiments. MS Ana includes its own decoy library generation algorithm which enables it to be used with any available target library. The library search and decoy generation are optimized for HCD fragmentation data. We demonstrate the performance of MS Ana on data from Orbitrap Fusion Lumos, Orbitrap Q Exactive, as well as timsTOF Pro mass spectrometers.

We validated MS Ana library search and decoy library generation using a data set of synthetic peptides. MS Ana was able to identify more spectra while maintaining lower FDR than the contemporary library search engine MSPepSearch. We also tested the performance of MS Ana using several configurations of publicly available human spectral libraries and showed the importance of library selection. In all tested cases MS Ana found more identifications than MSPepSearch or Sequest database search even when using smaller libraries or an entirely predicted spectral library. Finally, we tested MS Ana on data from single-shot data dependent acquisition experiments to demonstrate the performance in a typical biologically relevant experiment. Using a simple library search workflow MS Ana found on average 36.2% more spectrum matches and 4.1% more proteins than database search using state-of-the-art rescoring methods. Additionally, we repeated this benchmark on data from different experiments using a timsTOF Pro mass spectrometer. In this case, MS Ana found on average 9.4% more proteins than database search.

With the MS Ana Spectral Library Search Engine, we hope to provide a powerful and accessible software tool to the community to make spectral library search more readily available for mass spectrometry experiments. MS Ana can be downloaded free of charge from the IMP Web site (https://ms.imp.ac.at/index.php?action=ms-ana).

## Data Availability

You can download the MS Ana Spectral Library Search Engine free of charge from the IMP Web site (https://ms.imp.ac.at/index.php?action=ms-ana). MS Ana is currently available as a plugin node for the Proteome Discoverer software platform (Version 3.0, Thermo Fisher Scientific, Bremen) or as a standalone command line application. The MS Ana plugin node can be run with a free version of Proteome Discoverer which you can download from the ThermoFisher Web site (https://www.thermofisher.com/at/en/home/industrial/mass-spectrometry/liquid-chromatography-mass-spectrometry-lc-ms/lc-ms-software/multi-omics-data-analysis/proteome-discoverer-software.html). MS Ana makes use of the graphical user interface for workflow generation in Proteome Discover so users can easily set up searches with minimal bioinformatics knowledge. MS Ana was developed in C# and makes full use of multithreading operations for high performance.

## Supporting Information Available

Figure S1. Selection of synthetic peptide pools. Figure S2. Replication of validation test on synthetic peptide data. Figure S3. Spectral library search increases identifications for timsTOF data. Table S1. List of data sets. Table S2. List of spectral libraries. Table S3. Full results of synthetic peptide pool test with original pools. Table S4. Full results of synthetic peptide pool test with alternative pools. Table S5. Full results of library performance comparison. Table S6. Full results of single-shot benchmark test. Table S7. Full results of timsTOF benchmark test. (PDF)
Collection of the Proteome Discoverer workflow files used for data analysis. (ZIP)
