# Supplementary material for: MS Ana: Improving Sensitivity in Peptide Identification with Spectral Library Search
Source: J Proteome Res. 2023 Jan 23;22(2):462–70. doi: 10.1021/acs.jproteome.2c00658 (PMC9903325; doi:10.1021/acs.jproteome.2c00658)
Supplement: Supplementary file 1 — pr2c00658_si_001.pdf [file pr2c00658_si_001.pdf]

Supporting Information: MS Ana: Improving sensitivity in peptide identification with spectral library search

Sebastian Dorl,*,†,‡ Stephan Winkler,†,‡ Karl Mechtler,¶,§ and Viktoria Dorfer*,†

†*University of Applied Sciences Upper Austria, Bioinformatics Research Group, Softwarepark 11, 4232 Hagenberg, Austria*

‡*Johannes Kepler University Linz, Department of Computer Science, Altenbergerstraße 69, 4040 Linz, Austria*

¶*Research Institute of Molecular Pathology (IMP), Protein Chemistry, Campus-Vienna-Biocenter 1, 1030 Vienna, Austria*

§*Institute of Molecular Biotechnology (IMBA), Protein Chemistry, Vienna Biocenter (VBC), Dr. Bohr-Gasse 3, 1030 Vienna, Austria*

E-mail: sebastian.dorl@fh-hagenberg.at; viktoria.dorfer@fh-hagenberg.at

Phone: +43 (0) 50804 27145; +43 (0) 50804 22740

- Figure S1 .... Selection of synthetic peptide pools
- Figure S2 .... Replication of validation test on synthetic peptide data
- Figure S3 .... Spectral library search increases identifications for timsTOF data
- Table S1 .... List of datasets
- Table S2 .... List of spectral libraries
- Table S3 .... Full results of synthetic peptide pool test with original pools
- Table S4 .... Full results of synthetic peptide pool test with alternative pools
- Table S5 .... Full results of library comparison
- Table S6 .... Full results of single-shot benchmark test
- Table S7 .... Full results of timsTOF benchmark test

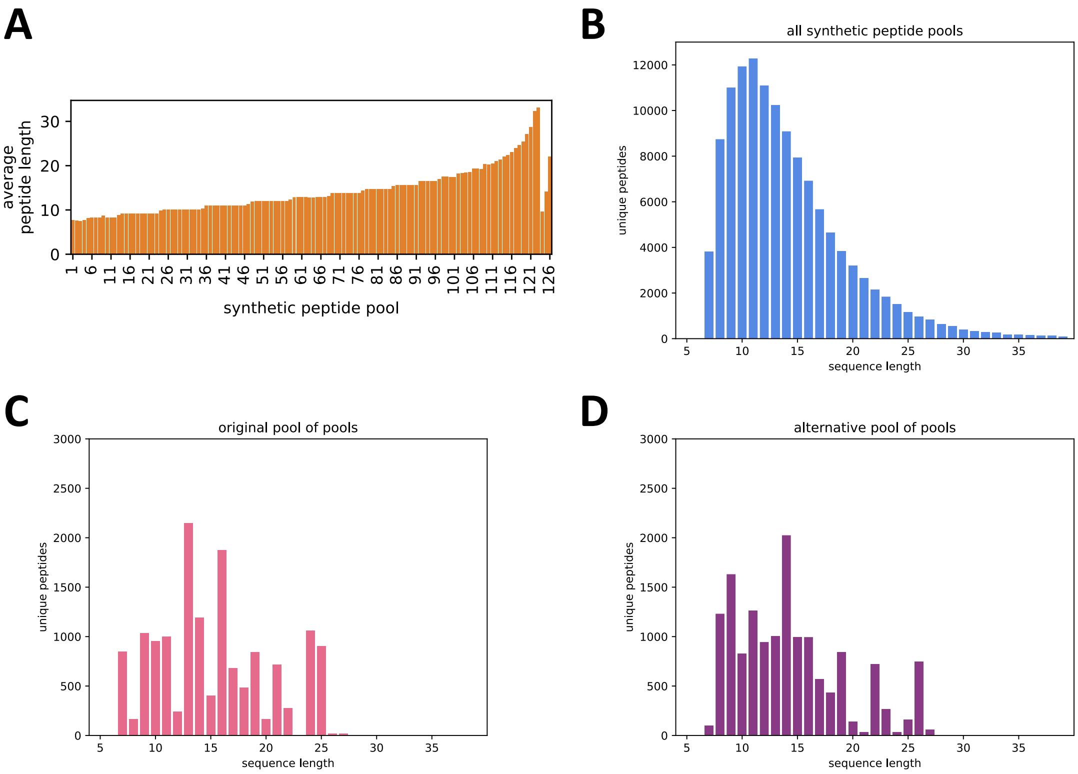

Figure S1: Selection of synthetic peptide pools. (A) Average length of sequences in proteotypic peptide pools of ProteomeTools (PXD004732). Because peptide length is uniform in each file we choose to create a ”pool of pools” for the test setup. (B) Distribution of sequence length for all peptides in the proteotypic set. Ideally, we should approximate the length distribution as best as possible in the benchmark test data. (C) Distribution of sequence length in the original selected pools for the benchmark test (see Supplementary Table S1). Set of pools was obtained by drawing 10,000 samples of 15 random pools and taking the result with minimal divergence between the distributions in B and C. (D) Distribution of sequence length in the alternative selected pools for a replication of the benchmark test (see Supplementary Figure S2 and Supplementary Table S4 for results). Analogously, this set of pools was obtained by drawing another 500,00 samples of 15 pools.

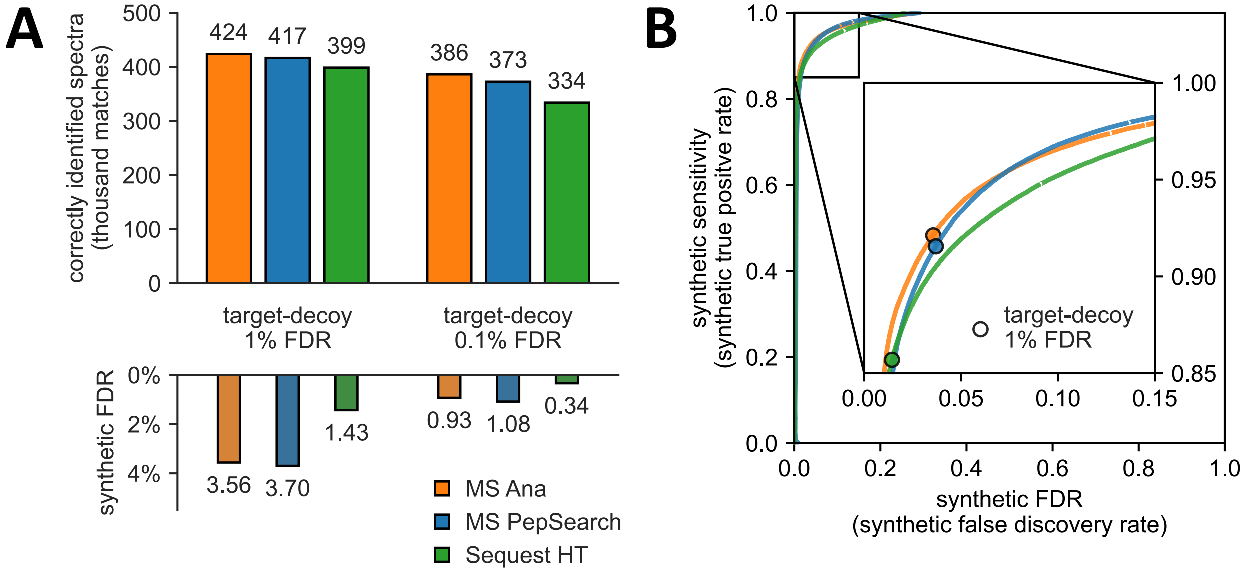

Figure S2: Spectral library search provides greater sensitivity during identification: Replication of validation tests on synthetic peptide data using an alternative set of peptides (see Supplementary Figure S1). Spectral library search and database search were tested on a combined HCD Orbitrap Fusion dataset of 778,674 spectra for a pool of 15,000 synthetic peptides obtained by Zolg et. al. (2017) (A) Comparison of correctly identified spectra. After validation by target-decoy search only matches that are in the list of synthetic peptides are retained as correct identifications and a new synthetic FDR is calculated accordingly (for full results see Supplementary Table S4). (B) Comparison of synthetic FDR and sensitivity for the search results. True positives are those spectra which were correctly identified from the list of synthetic peptide pool sequences. The specific threshold for target-decoy FDR 1% is highlighted.

Table S1: List of datasets.

dataset	spectra	repository ID	file name	results
original pool 1/15	45,687	PXD004732	01625b_GB1-TUM_first_pool_2_01_01-3xHCD-1h-R1.raw	Figure 1,Table S3
original pool 2/15	54,561	PXD004732	01625b_GB4-TUM_first_pool_26_01_01-3xHCD-1h-R1.raw	Figure 1,Table S3
original pool 3/15	54,393	PXD004732	01625b_GB6-TUM_first_pool_42_01_01-3xHCD-1h-R1.raw	Figure 1,Table S3
original pool 4/15	51,405	PXD004732	01625b_GG3-TUM_first_pool_23_01_01-3xHCD-1h-R1.raw	Figure 1,Table S3
original pool 5/15	55,311	PXD004732	01650b_BB5-TUM_first_pool_76_01_01-3xHCD-1h-R2.raw	Figure 1,Table S3
original pool 6/15	54,249	PXD004732	01650b_BC6-TUM_first_pool_85_01_01-3xHCD-1h-R2.raw	Figure 1,Table S3
original pool 7/15	53,592	PXD004732	01650b_BD6-TUM_first_pool_86_01_01-3xHCD-1h-R2.raw	Figure 1,Table S3
original pool 8/15	55,566	PXD004732	01650b_BF3-TUM_first_pool_64_01_01-3xHCD-1h-R2.raw	Figure 1,Table S3
original pool 9/15	55,974	PXD004732	01650b_BG3-TUM_first_pool_65_01_01-3xHCD-1h-R2.raw	Figure 1,Table S3
original pool 10/15	52,407	PXD004732	01650b_BG7-TUM_first_pool_97_01_01-3xHCD-1h-R2.raw	Figure 1,Table S3
original pool 11/15	48,126	PXD004732	01709a_GB3-TUM_first_pool_116_01_01-3xHCD-1h-R1.raw	Figure 1,Table S3
original pool 12/15	54,528	PXD004732	01709a_GB4-TUM_first_pool_125_01_01-3xHCD-1h-R1.raw	Figure 1,Table S3
original pool 13/15	48,549	PXD004732	01709a_GC3-TUM_first_pool_117_01_01-3xHCD-1h-R1.raw	Figure 1,Table S3
original pool 14/15	48,201	PXD004732	01709a_GE2-TUM_first_pool_111_01_01-3xHCD-1h-R1.raw	Figure 1,Table S3
original pool 15/15	48,114	PXD004732	01709a_GG1-TUM_first_pool_105_01_01-3xHCD-1h-R1.raw	Figure 1,Table S3
alternative pool 1/15	48,192	PXD004732	01625b_GB2-TUM_first_pool_10_01_01-3xHCD-1h-R1.raw	Figure S2, Table S4
alternative pool 2/15	50,787	PXD004732	01625b_GE2-TUM_first_pool_13_01_01-3xHCD-1h-R1.raw	Figure S2, Table S4
alternative pool 3/15	52,227	PXD004732	01625b_GB3-TUM_first_pool_18_01_01-3xHCD-1h-R1.raw	Figure S2, Table S4
alternative pool 4/15	50,943	PXD004732	01625b_GC5-TUM_first_pool_35_01_01-3xHCD-1h-R1.raw	Figure S2, Table S4
alternative pool 5/15	53,067	PXD004732	01650b_BB1-TUM_first_pool_44_01_01-3xHCD-1h-R2.raw	Figure S2, Table S4
alternative pool 6/15	55,113	PXD004732	01650b_BF1-TUM_first_pool_48_01_01-3xHCD-1h-R2.raw	Figure S2, Table S4
alternative pool 7/15	55,452	PXD004732	01650b_BA4-TUM_first_pool_67_01_01-3xHCD-1h-R2.raw	Figure S2, Table S4
alternative pool 8/15	55,470	PXD004732	01650b_BE4-TUM_first_pool_71_01_01-3xHCD-1h-R2.raw	Figure S2, Table S4
alternative pool 9/15	56,616	PXD004732	01650b_BH4-TUM_first_pool_74_01_01-3xHCD-1h-R2.raw	Figure S2, Table S4
alternative pool 10/15	54,075	PXD004732	01650b_BH5-TUM_first_pool_82_01_01-3xHCD-1h-R2.raw	Figure S2, Table S4
alternative pool 11/15	53,010	PXD004732	01650b_BA7-TUM_first_pool_91_01_01-3xHCD-1h-R2.raw	Figure S2, Table S4
alternative pool 12/15	52,407	PXD004732	01650b_BG7-TUM_first_pool_97_01_01-3xHCD-1h-R2.raw	Figure S2, Table S4
alternative pool 13/15	48,114	PXD004732	01709a_GG1-TUM_first_pool_105_01_01-3xHCD-1h-R1.raw	Figure S2, Table S4
alternative pool 14/15	46,554	PXD004732	01709a_GG2-TUM_first_pool_113_01_01-3xHCD-1h-R1.raw	Figure S2, Table S4
alternative pool 15/15	46,647	PXD004732	01709a_GD3-TUM_first_pool_118_01_01-3xHCD-1h-R1.raw	Figure S2, Table S4
library test REP1	118,074	PXD028735	LFQ_Orbitrap_DDA_Human_01.raw	Figure 2, Table S4
library test REP2	115,494	PXD028735	LFQ_Orbitrap_DDA_Human_02.raw	Figure 2, Table S4
library test REP3	114,053	PXD028735	LFQ_Orbitrap_DDA_Human_03.raw	Figure 2, Table S4
ssDDA test REP1	163,361	PXD023587	200807_mcf7_50ug_2h_R1.raw	Figure 3, Table S5
ssDDA test REP2	163,339	PXD023587	200807_mcf7_50ug_2h_R2.raw	Table S5
ssDDA test REP3	163,014	PXD023587	200807_mcf7_50ug_2h_R3.raw	Table S5
timsTOF test REP1	179,527	MSV000087476	Azo_30m_ext1_1p5times_inc_Slot1-29_1_823.d	Figure S2, Table S6
timsTOF test REP2	175,657	MSV000087476	Azo_30m_ext2_1p5times_inc_Slot1-30_1_824.d	Table S6
timsTOF test REP3	186,771	MSV000087476	Azo_30m_ext3_1p5times_inc_4_Slot1-31_1_834.d	Table S6

Table S2: List of spectral libraries.

organism	name	source	spectra	peptides	results
Human	KB 2.0.15, in vivo, HCD only	MassIVE KB v2 - full releases	2,140,865	1,055,165	Figures: 2, 3, S3; Tables S5, S6, S7
Human	KB 2.0.15, synthetic	MassIVE KB v2 - full releases	1,662,275	889,204	Figures: 1, 2, S2; Tables S3, S4, S5
Human	”Library 1 (best)”	NIST Human HCD Libraries	398,373	257,122	Figure 2, Table S5
Human	”Library 2 (good)”	NIST Human HCD Libraries	200,477	108,066	Figure 2, Table S5
Arabidopsis	Library of Unmodified Peptides	ProteomicsDB	482,646	328,000	Figure 2, Table S5
Human	Predicted Uniprot Swissprot	INFERYS in PD 3.0	5,253,666	5,253,666	Figure 2, Table S5

Table S3: Full results of synthetic peptide pool test with original set of pools (Figure 1).

search engine	FDR%	syn. FDR%	PSMs	correct PSMs	syn. TPR%	syn. FPR%
MS Ana	5.0	9.85	491,397	443,007	96.97	26.94
MS Ana	1.0	3.04	436,704	423,423	92.68	07.39
MS Ana	0.1	0.89	395,845	392,327	85.88	01.96
MSPepSearch	5.0	10.81	494,548	441,064	97.49	28.64
MSPepSearch	1.0	3.29	431,027	416,832	92.13	07.60
MSPepSearch	0.1	0.95	371,274	367,759	81.29	01.88
Sequest	5.0	5.88	454,734	427,990	92.86	17.04
Sequest	1.0	1.33	396,614	391,358	84.91	03.35
Sequest	0.1	0.26	308,694	307,900	66.81	00.51

Table S4: Full results of synthetic peptide pool test with alternative set of pools (Supplementary Figure S2).

search engine	FDR%	syn. FDR%	PSMs	correct PSMs	syn. TPR%	syn. FPR%
MS Ana	5.00	10.82	499,545	445,505	96.84	29.61
MS Ana	1.00	3.56	439,424	423,778	92.11	08.57
MS Ana	0.10	0.93	389,782	386,164	83.94	01.98
MSPepSearch	5.00	11.13	497,741	442,322	97.20	29.66
MSPepSearch	1.00	3.70	432,579	416,594	91.55	08.55
MSPepSearch	0.10	1.08	376,695	372,619	81.89	02.18
Sequest	5.00	6.35	462,068	432,734	93.01	18.30
Sequest	1.00	1.43	404,454	398,665	85.69	03.61
Sequest	0.10	0.34	335,062	333,932	71.78	00.70

Table S5: Full results of library performance comparison (1% FDR). (Figure 2)

search space	search engine	rep.	PSMs	PSMs with Percolator
MassIVE HCD	MS Ana	1	73,303	79,221
MassIVE HCD	MSPepSearch	1	67,552	76,712
MassIVE HCD	MS Ana	2	70,604	77,004
MassIVE HCD	MSPepSearch	2	65,408	74,132
MassIVE HCD	MS Ana	3	70,611	77,021
MassIVE HCD	MSPepSearch	3	64,730	74,209
MassIVE HCD+Syn	MS Ana	1	73,390	79,899
MassIVE HCD+Syn	MSPepSearch	1	66,204	76,651
MassIVE HCD+Syn	MS Ana	2	70,277	77,453
MassIVE HCD+Syn	MSPepSearch	2	63,648	74,018
MassIVE HCD+Syn	MS Ana	3	70,562	77,494
MassIVE HCD+Syn	MSPepSearch	3	63,704	73,853
NIST Best	MS Ana	1	56,253	62,673
NIST Best	MSPepSearch	1	46,132	58,971
NIST Best	MS Ana	2	54,239	60,915
NIST Best	MSPepSearch	2	44,614	57,018
NIST Best	MS Ana	3	54,351	61,041
NIST Best	MSPepSearch	3	44,841	56,980
NIST Best+Good	MS Ana	1	58,188	65,053
NIST Best+Good	MSPepSearch	1	47,835	61,017
NIST Best+Good	MS Ana	2	56,008	63,285
NIST Best+Good	MSPepSearch	2	45,920	58,927
NIST Best+Good	MS Ana	3	56,220	63,579
NIST Best+Good	MSPepSearch	3	46,271	58,813
NIST Best+Ara	MS Ana	1	55,222	63,130
NIST Best+Ara	MSPepSearch	1	44,364	58,730
NIST Best+Ara	MS Ana	2	53,075	61,324
NIST Best+Ara	MSPepSearch	2	42,526	56,632
NIST Best+Ara	MS Ana	3	53,421	61,572
NIST Best+Ara	MSPepSearch	3	42,712	56,773
Predicted Swissprot	MS Ana	1	58,304	63,205
Predicted Swissprot	MSPepSearch	1	40,180	59,870
Predicted Swissprot	MS Ana	2	55,479	60,599
Predicted Swissprot	MSPepSearch	2	38,220	57,433
Predicted Swissprot	MS Ana	3	55,603	61,023
Predicted Swissprot	MSPepSearch	3	37,574	57,491
Uniprot Swissprot	Sequest	1	37,906	58,164
Uniprot Swissprot	Sequest INFERYS	1	-	60,892
Uniprot Swissprot	Sequest	2	36,781	55,571
Uniprot Swissprot	Sequest INFERYS	2	-	58,608
Uniprot Swissprot	Sequest	3	36,876	55,748
Uniprot Swissprot	Sequest INFERYS	3	-	58,718

Table S6: Full results of single-shot benchmark test (1% FDR). (Figure 3)

replicate	search engine	Percolator	INFERYS	PSMs	proteins (PD)
1	MS Ana			89,919	-
1	MS Ana	yes		96,875	7,228
1	MSPepSearch			83,300	-
1	MSPepSearch	yes		91,943	7,097
1	Sequest			48,602	-
1	Sequest	yes		68,809	-
1	Sequest	yes	yes	71,280	6,930
2	MS Ana			89,787	-
2	MS Ana	yes		96,977	7,230
2	MSPepSearch			82,829	-
2	MSPepSearch	yes		91,741	7,055
2	Sequest			48,488	-
2	Sequest	yes		68,829	-
2	Sequest	yes	yes	71,031	6,910
3	MS Ana			88,689	-
3	MS Ana	yes		96,127	7,168
3	MSPepSearch			82,515	-
3	MSPepSearch	yes		91,123	7,039
3	Sequest			47,762	-
3	Sequest	yes		68,190	-
3	Sequest	yes	yes	70,548	6,932

Table S7: Full results of timsTOF benchmark test (1% FDR). (Figure S3)

replicate	search engine	Percolator	PSMs	proteins (PD)
1	MS Ana		62,361	-
1	MS Ana	yes	68,859	4,334
1	MSPepSearch		64,684	-
1	MSPepSearch	yes	68,811	4,296
1	Sequest		62,005	-
1	Sequest	yes	68,252	3,879
2	MS Ana		64,509	-
2	MS Ana	yes	68,347	4,394
2	MSPepSearch		66,354	-
2	MSPepSearch	yes	72,468	4,248
2	Sequest		68,403	-
2	Sequest	yes	72,355	3,891
3	MS Ana		27,779	-
3	MS Ana	yes	47,858	4,307
3	MSPepSearch		29,728	-
3	MSPepSearch	yes	48,819	4,184
3	Sequest		28,012	-
3	Sequest	yes	50,249	3,860

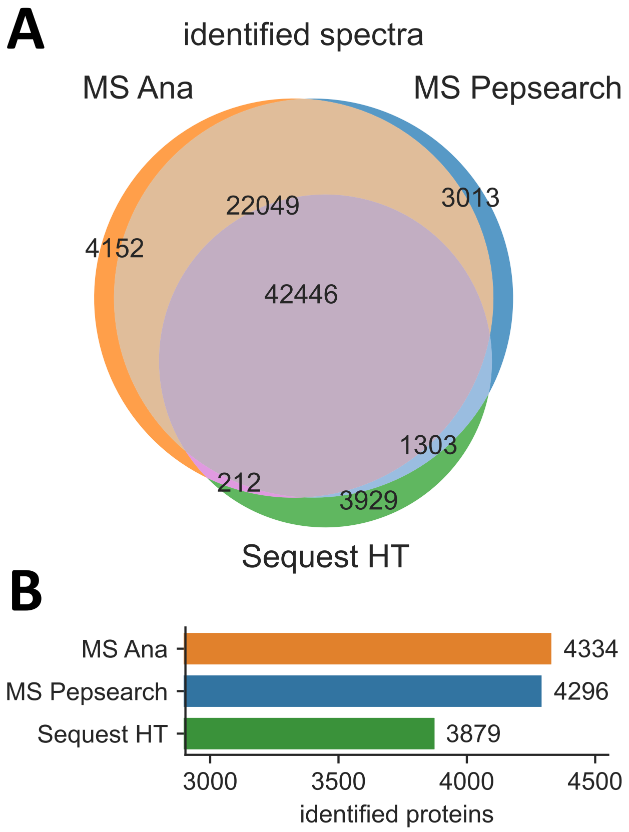

Figure S3: Spectral library search increases identifications for timsTOF data. Benchmark of identification algorithms on data from 20 mg of human LV cardiac tissue measured on a Bruker timsTOF Pro by Aballo et. al. (2021). Search results of the first replicate comparing spectral library search using MS Ana (orange) or MSPepSearch (blue) on MassIVE Human HCD library with database search using Sequest (green) on Uniprot Swissprot (INFERYS rescoring is not available for this type of data). (A) Venn diagram showing the overlap of peptide-to-spectrum matches between the different identification algorithms (1% FDR using Percolator) (B) Number of identified proteins from result (1% Protein-level FDR applied after filtering by Percolator). For full results see Supplementary Table S7.
